# The transition of general practice into an academic discipline: tracing the origins through the first four professors in general practice/family medicine

**DOI:** 10.1080/02813432.2024.2335537

**Published:** 2024-04-16

**Authors:** Jørund Straand, Niek de Wit

**Affiliations:** a General Practice Research Unit (AFE), Department of General Practice/Family Medicine, University of Oslo, Oslo, Norway; b Department of General Practice, Julius Center of Health Sciences and Primary Care, University Medical Center (UMC) Utrecht, Utrecht, The Netherlands

**Keywords:** Family medicine/history, university departments/professors, Edinburgh, Utrecht, Western Ontario, Oslo

## Abstract

Being the ‘mother’ of most clinical specialties, general practice is as old as medicine itself. However, as a recognized academic discipline within medical schools, general practice has a relatively short life span. A decisive step forward was taken in 1956 when the University of Edinburgh established its Department of General Practice, and appointed the world’s inaugural professor in the field in 1963. During the 1960s, the pioneering move in Edinburgh was followed by universities in the Netherlands (University of Utrecht), Canada (Western University, Ontario), and Norway (University of Oslo), marking the beginning of global academic recognition for general practice/family medicine. Despite its critical role in healthcare, the academic evolution of general practice has been sparingly documented, with a notable absence of comprehensive accounts detailing its integration into medical schools as an independent discipline with university departments and academic professors. Last year (2023) marked the 60th anniversary of Dr. Richard Scott’s historic appointment as the first professor of General Practice/Family Medicine. Through the lens of the first four professors appointed between 1963 and 1969, we explore the ‘birth’ of general practice to become an academic discipline. In most western countries of today, general practice has become a recognized medical discipline and an important part of the medical education. But many places, this development is lagging behind. The global shaping of general practice into an academic discipline is therefore definitively not completed.

## Introduction

In the nineteenth century, hospitals were few and medical practice was by large clinical work in primary care. Physicians working in single-handed practices saw their patients during office consultations and home visits. In many respects, general practice may be regarded as the ‘mother’ of most other clinical specialties in medicine. Up to, say, World War II (WWII), the most common image of a doctor in western countries, was that of a doctor in primary care, the private practitioner.

From mid-nineteenth century onwards, the center of gravity of medicine gradually shifted from general practice to the hospital sector. After WWII, more physicians were working in hospitals than in primary care. Across the world, hospital medicine developed into an increasing number of different specialties with corresponding academic units at the medical faculties and teaching hospitals. This development was also reflected in the clinical curriculum for medical students. At the same time, general practice was lagging behind. General practitioners (GPs) were isolated in single-handed practices with lack of professional leadership and development. They were represented neither in education nor in research, and academic departments of general practice were non-existent. It became commonplace to regard GPs as second-class doctors. Diminishing number of GPs and increasing workload for those remaining in practice created a vicious circle. Many old GPs dreamt of retirement while young doctors chose other branches of medicine. No doubt, general practice at the time was in a crisis. Because medicine had become more and more specialized, some even considered general practice such an outdated branch of medicine that it preferably should be replaced by modern outpatient clinics staffed with hospital specialists. For example, the Swedish director general of the National Board of Health and Welfare during the 1960s, Bror Rexed (1914–2002) argued that GPs only represented a temporary solution for some remote districts. Neither he nor his family members would choose a GP as long as hospital specialists were available! [1]

However, the bad situation for general practice during the 1950-ies and early 1960s also caused a number of GPs to stand up and fight for their profession as a matured medical discipline. They argued that general practice should be the core medical specialty in primary care. This demanded that general practice had to become part of the curriculum at the medical schools. This paved the way for the development of general practice/family medicine (GP/FM) as an academic discipline with university departments, headed by professors in general practice, with under- and postgraduate teaching and a research agenda.

As GPs, whether in clinical practice, in academia or in both, we should know our history, and how our profession and discipline developed. The aim of this article is to briefly describe aspects of the ‘pregnancy and birth’ of general practice as an academic discipline. During the years 1963–1969, the first four universities in the world founded academic departments with professors in GP/FM. The story starts in the UK, moves on to the Netherlands, Canada, and ends in Norway.

## The United Kingdom

### The National Health Service and the Collins report

Shortly after WWII, a young Australian, Joseph Silver Collings (1918–1971), worked as a research fellow at the US Harvard School of Public Health. Being a physician himself, he had previously worked as a GP in New Zealand. At Harvard he had studied primary care in parts of the US and Canada. In 1948 he received a grant from the Nuffield Trust to undertake a review of UK general practice under the newly formed National Health Service (NHS). To do this, Collins visited a random selection of 55 general practices (104 GPs) throughout England and Scotland. His observations were summed up in the so-called Collins report published in 1950 in the Lancet spanning over 30 pages [2].

Collins’ description of UK general practice was indeed harsh: *‘General Practice is accepted as being something specific, without anyone knowing what it really is. …. While other branches of medicine have progressed and developed, general practice, instead of developing concurrently, has adapted itself to the changing patterns; and sometimes this adaptation has in fact been regression. There are no real standards for general practice. What the doctor does, and how he does it, depends almost wholly on his own conscience’.*

In his report, Collins described ill-equipped and dirty practices and GPs with outdated knowledge. *‘Some conditions of general practice are bad enough to change a good doctor to a bad doctor within a very short time. These very bad conditions are to be found in industrial areas’.* His conclusion was that ‘*the over-all state of general practice is bad and still deteriorating’* [2].

### The Royal College of general practitioners

The publication of the Collins report attracted tremendous attention in the UK medical community. For some GPs it served as a wake-up call. Shortly after (1951), the GPs Fraser Rose and John Hunt, published a letter to the Lancet calling for a national college for GPs [3]:

There is a College of Physicians, a College of Surgeons, a College of Obstetricians and Gynaecologists, a College of Nursing, a College of Midwives, and a College of Veterinary Surgeons, all of them Royal Colleges; there is a College of Speech Therapists and a College of Physical Education; but there is no College or Academic Body to represent primarily the interests of the largest group of medical personnel in this country - the 20,000 general practitioners.

Also inspired by the American college for general practice (The American academy of Family Physicians, established in 1947), the challenge put forward by Rose and Hunt accelerated a process that resulted in the foundation of The Royal College of General Practitioners (RCGP) in 1952. One of the first priorities for the RCGP was to have its own journal. The Journal of RCGP (Now: British Journal of General Practice) launched its first issue in 1953.

#### The James Mackenzie chair in general practice at the University of Edinburgh

After the foundation of the NHS, the University of Edinburg converted a dispensary (a primary health care center where medicines were prepared and given out) to a teaching practice for medical students. Here, students were given an optional opportunity to observe and learn general practice which otherwise was not part of their curriculum.

By the turn of the century, the Scottish GP James Mackenzie (1853–1925), had done groundbreaking cardiovascular research in his general practice in Burnley, England.^4^ In 1963, his daughter, Ms. Dorothea Mackenzie, donated funds to the University of Edinburgh to establish a professorship in general practice in her father’s memory. This external funding paved the way for the first general practice professor in the world, the James Mackenzie chair in general practice. The first professor was appointed the same year: Richard (‘Dick’) Scott (1914–1983), Figure 1. Ever since 1946, Scott had been a lecturer and reader at the Department of Public Health and Social Medicine, and he had chaired the General Practice Teaching Unit for medical students in Edinburgh since 1948. During the 1950s Richard Scott wrote several articles about education in general practice, and about the Edinburgh general practice teaching unit [5].

**Figure 1.**
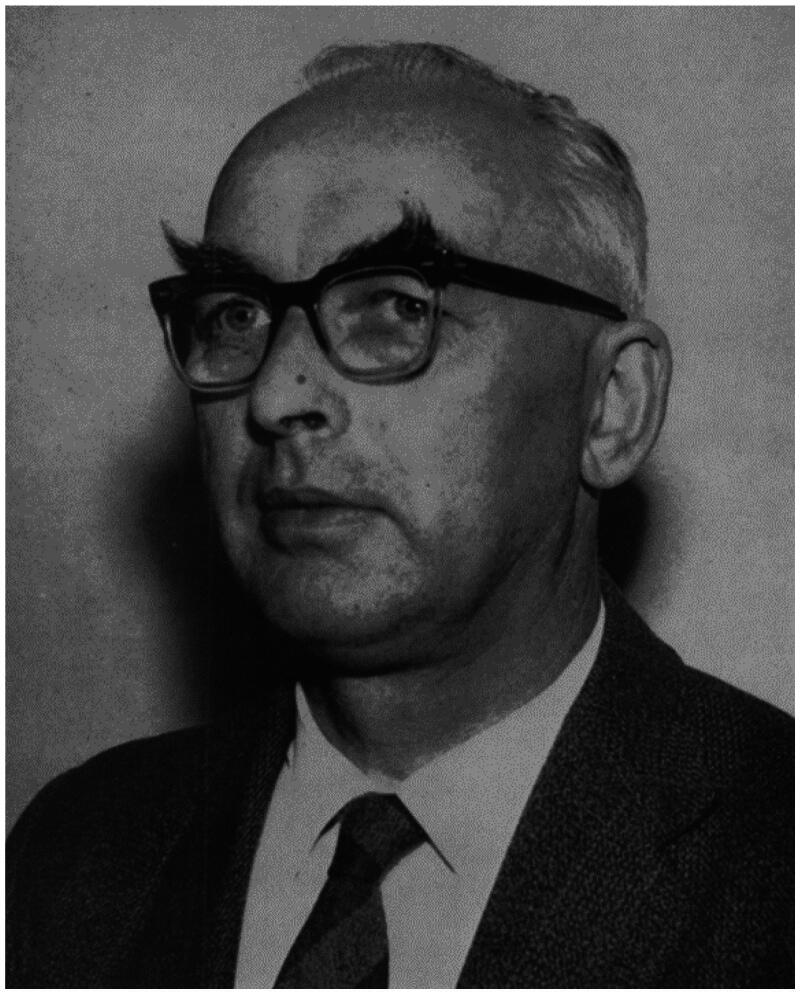
Richard (‘Dick’) Scott (1914–1983). In 1963, he became the first professor in General Practice/Family Medicine in the world at the University of Edinburgh, Scotland.

At the time when Richard Scott entered the chair in Edinburgh, he published a chapter in a WHO report about ‘The work of the physician in the community’ [6]. In this article, Scott described the essentials of general practice explaining why it should be a clinical specialty:

General practice is primarily the area of undifferentiated medicine…where society and medicine meet. (…) General practice is not inferior or superior to specialist practice; it is a different field of medicine. (…) There are two characteristics of general practice which distinguish the general practitioner from any other medically qualified person: the patient has direct and unqualified access to the general practitioner; the general practitioner provides continuity of care. (…)

The incurable, the uncooperative, the impossible patients before him today will still be his patients tomorrow and the day after; and the general practitioner and his patient have to learn to live with the problem. In addition to these difficult clinical challenges, the general practitioner is also exposed to a wide range of social and personal problems which his patients bring to him and for which he sees no certain answer.

He also (…) acts as a link or an integrator between general medical practice and the services provided by his hospital or specialist colleagues. [6]

When Richard Scott retired at the age of 65 in 1979, his successor Professor John Howie (1937–) quite simply said of him that Richard Scott had taken academic general practice from nowhere to somewhere [7]. 69 years old, Scott died in 1983. In the obituary printed in the Lancet, Scott was described as *‘an ideal of the quiet revolutionary*’, with a ‘*strength of will and purpose ….behind the deceptively quiet exterior…. Without his own strong-minded sense of mission and dogged determination, however, we would not have reached so far so fast in academic general practice.*’ [8]

In Scotland, three more chairs in general practice followed Edinburgh between 1970 and 1972^9^. The University of Manchester was first in England to have a department with a chair (1972). It was not until 1995 that all UK medical schools had general practice departments.

## Utrecht, The Netherlands (1966)

As in the UK, GPs in the Netherlands had been working as GPs (*algemeen arts* or *huisarts)* in a rather unorganized way for many years. In the late nineteenth century, there were more than 2000 ‘general physicians’ and only 37 (hospital) specialists. From the early twentieth century specialist medicine developed, and the tension between specialist medicine and general practice grew. After WWII, a poor financial situation and high workload meant that general practice was not highly valued either by the public, the medical community or the medical students.

**Figure 2.**
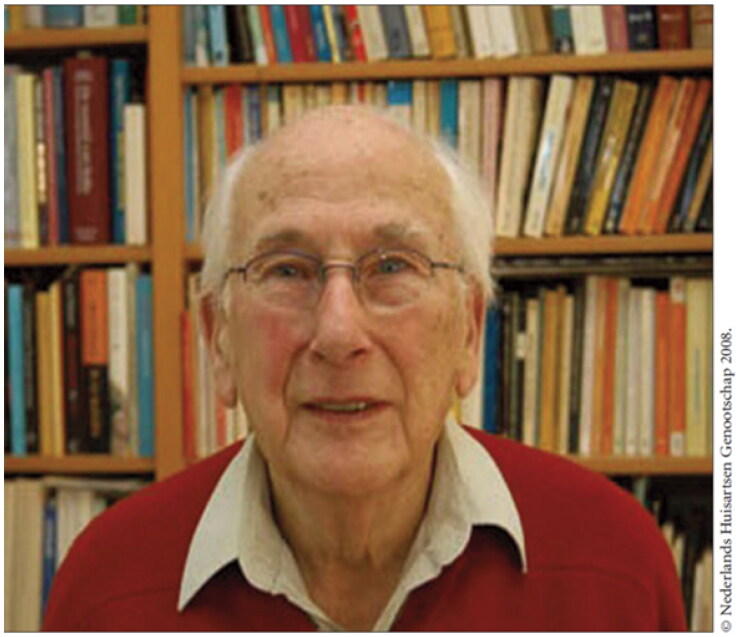
Jan van Es (1921–2008). In 1966, he accepted a post of extraordinary professor in General Practice at Utrecht University, The Netherlands. He was the second professor in General Practice/Family Medicine in the world.

In 1950, Just Buma, a GP from a small community in the West of Holland, published his PhD thesis *The GP and his patient*, which was very influential [10]. As from 1947 he had built up a morbidity registry of the patients in his practice. He found out that for half of the complaints in his daily work, no somatic cause could be found. Therefore, Buma underlined in his thesis the need for a more holistic and integrated approach in medicine. In 1956 he joined a group of GPs (including among others, Hein Hogerzeil, Frans Huygen and Jan van Es) to establish the Dutch College of General Practitioners (*Nederlands Huisarts Genootschap, NHG*.) Jan van Es (1921–2008; Figure 2), was a GP working in Apeldoorn and just about to start his own doctoral research project investigating families and children with mental disability [11]. Having published his thesis in 1959, van Es became deputy chair and member of the Committee for Scientific Research of the Dutch college. Through his position in the college, he developed a plan for a Dutch Institute of General Practitioners, which was officially opened in 1964 by Her Highness the Queen. Jan van Es became the first director of the institute which later became the Netherlands Institute for Health Services Research (NIVEL). His work at NIVEL resulted in Utrecht University offering him a new post of extraordinary professor in general practice in 1966. This was the second professorship in general practice after Professor Richard Scott at Edinburgh.

Van Es continued working part time as a GP and to do own research on ill health and social conditions [12]. In 1968, the extraordinary chair of Jan van Es was converted into a regular professorship, and the Utrecht University inaugurated the University Institute for General practice. At the Utrecht university, van Es developed the postgraduate general practice training program, including skills training, pharmacotherapy, communication and interviewing techniques, and laboratory testing use in general practice. Niek de Wit, current professor of general practice at Utrecht University and coauthor of this article, underlines the importance that Jan van Es had for the installment of the vocational training:’ *It required vision and analytic competencies to translate the clinical and communication skills that were considered vital for general practice into a training program, and leadership to get this implemented nationwide.’*

Jan van Es also wrote a textbook on general practice which was widely used in medical training in the Netherlands for many years [13].

In 1974 van Es helped to establish the international Leeuwenhorst Group which made a lasting contribution to defining general practice in Europe. Their work is now continued by The European Academy of Teachers in General Practice (EURACT) under the umbrella of WONCA Europe.

In 1982, van Es became editor in chief of the Dutch Medical Association’s journal and he took up a professorship with Amsterdam’s Free University promoting collaboration between general practitioners and hospital consultants.

In his semi-retirement from 1993 onwards, van Es directed a postgraduate training program for GPs in the former communist countries Poland, Hungary, and Romania.

In 2006 Van Es wrote his memoirs, *Half a Century of General Practice: from Trade to Profession* [14]. Here he underlined the importance of continuity of care in general practice for building up of diagnostic and therapeutic knowledge about the patients.

His recipe for strengthening general practice as a scientific discipline was to set up a national professional body, develop academic scientific centers, and invest in the political process required for sustainable professional development. One of the leading principles of Van Es, was the interaction between research and practice. In his view evidence-based practice had to rely on practice based evidence: *‘We have to have research and data on practice, - primary care must be evidence-based.’* [15]

Today van Es is remembered as the Godfather of Dutch general practice and as a pioneer of evidence based primary care. He laid the fundament for the current academic departments of general practice at all the seven universities in the Netherlands.

Jan Van Es died on 28 June 2008 at the age of 86.

## Ontario, Canada (1968)

**Figure 3.**
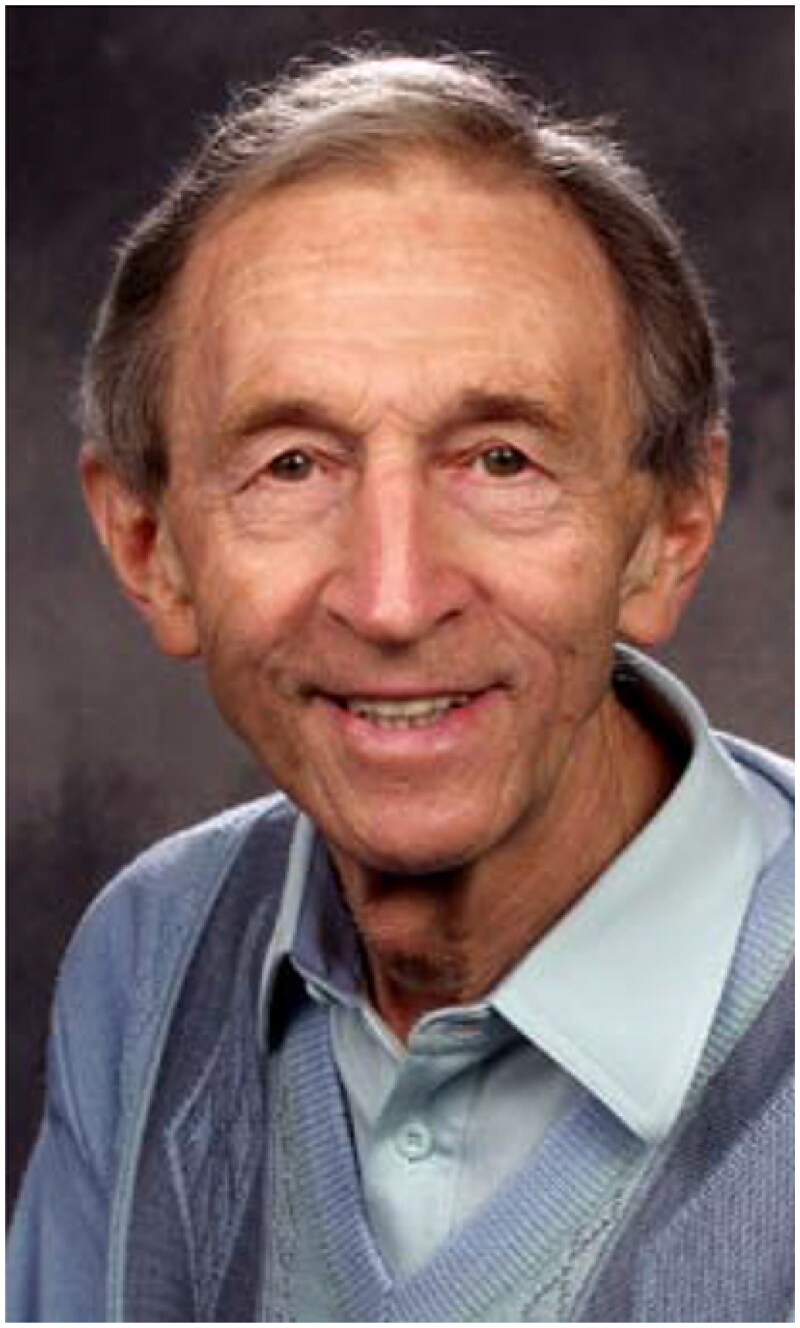
Ian McWhinney (1926–2012). He was ‘headhunted’ from England to Western University in London Ontario, Canada to become their first professor in General Practice/Family Medicine. He entered the chair in May 1968 as the third professor in the discipline in the world.

Ian McWhinney (1926–2012) (Figure 3) had been practicing as a GP for 13 years in Burnley, England, when he in 1964 published his first book, *‘The Early Signs of Illness: Observations in General Practice’* [16]. In the foreword, Professor Richard Scott stated that *‘The author of this book is clearly a person who is fascinated and even excited by the clinical challenges which confront him in family medicine.’* McWhinney himself sat the tune by the following opening phrase: *‘The recognition of disease in its earliest stages calls for clinical expertise of the highest order. This is a skill which cannot be learned in hospital’* [16].

In 1964 McWhinney gained a Nuffield Traveling Fellowship to study family medicine in the US and Canada. This resulted in two papers in the Lancet, the first of which was about general practice as an academic discipline [17]. McWhinney listed four criteria which had to be fulfilled for general practice to become an academic discipline: (1) A unique field of action. (2) A defined body of knowledge. (3) An active area of research. (4) A training which is intellectually rigorous [17]. In the second paper, McWhinney discussed the role of the primary physician in a comprehensive health service [18]. Among others, he highlighted defects in the current health care system such as the unhappy separation of general practice from the main body of medicine. According to McWhinney, family medicine should step up and take its place as one of the special disciplines in medicine.

In 1968, McWhinney was invited to Western University Ontario in Canada to become their first professor in family medicine. Here, he soon gained international reputation as an influential thinker and philosopher in family medicine. His *‘A textbook of family medicine*’, introduced the patient-centered clinical method which has ever since been influential for GPs worldwide [19]. McWhinney presented the terms ‘patient’s agenda and doctor’s agenda’ and the need for their integration during the consultation. He wrote: *‘Illness is what you have when you go to the doctor; disease is what you have when you’ve seen the doctor. The aim of the patient-centered method is to understand the illness and, whenever possible, to diagnose the disease.’*

McWhinney held the position as professor in family medicine at Western University until he stepped down from the chair in 1987. He received honorary degrees from the University of Oslo and Western University in Canada. In 2006, he was inducted into the Canadian Medical Hall of Fame. At the age of 88, Ian McWhinney died in 2012.

Ian McWhinney is remembered as the founding father of family medicine in Canada. Being both an influential thinker and the third professor in GP/FM in the world, he is clearly also well qualified for a prominent place in the global hall of fame for general practice.

## Oslo, Norway (1968)

After WWII, the situation in Norwegian general practice was quite similar to the situation in many other western countries at that time. Despite an increasing population and in total number of physicians, the number of GPs went down. GPs were working in the shadow of their hospital colleagues, isolated from the medical community, and experiencing increasing workload (on average 63 h/week in 1966) and loss of status [1]. Hospital specialists and health authorities commonly considered GPs to represent low quality medicine. Few young doctors chose a future career in general practice.

The national health authorities had their priority on building up a modern hospital sector while general practice was neglected, especially GPs in private practice. By the early 1960s, the situation was dire, the country faced an imminent risk for total breakdown of the primary health care system.

During the 1950s, the GP Bent Guttorm Bentsen (1926–2008) had combined clinical work in his large rural practice with research aiming to describe the content of general practice. This resulted in 14 articles published during 1961–1966 [20]. The articles contributed to highlighting the uniqueness of general practice, while at the same time discussing many of the central methodological problems in general practice research [20]. In 1966 he submitted his work to The Medical Faculty to be assessed as a doctoral thesis. However, the work was refused, which in retrospect has been judged as a scandal and a shame for the Faculty [21]. If his doctoral thesis had been approved, Bentsen had clearly been a strong candidate for the professorship in Oslo in 1968. In 1970 his work was published in English as the book *Illness in general practice* [22]. Inspired by his own research, Bentsen participated in forming an activist group (‘the push and pull group’) within the Norwegian Association of General Practitioners to fight for making general practice a recognized medical speciality [21]. The time was ripe - in the 1960s there was ‘something in the air’, a zeitgeist of decentralization and bottom-up democracy. The medical students wanted more everyday medicine into their curriculum. The ‘push and pull group’ argued that an essential measure for rescuing general practice from a silent death, was to make general practice part of the curriculum for all medical students. To achieve this, general practice had to be restored from just a trade and a field of practice to become a recognized medical discipline. Consequently, medical schools had to set up university departments with professors in GP/FM. After some time, this became the official policy of the Norwegian Medical Association (NMA) too.

**Figure 4.**
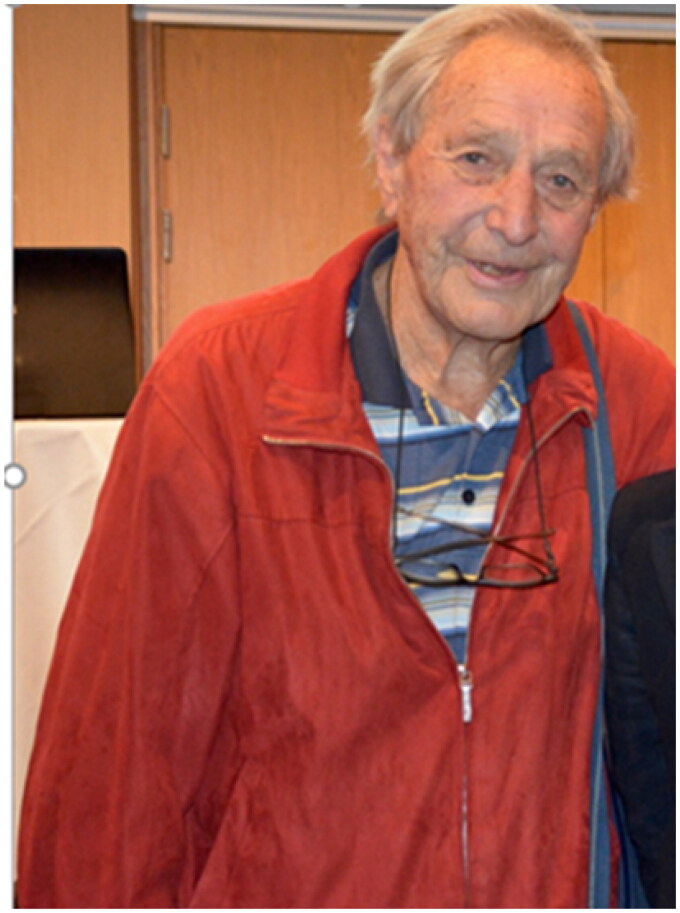
Christian Fredrik Borchgrevink (1924–). He accepted to be acting professor and head of the new Institute of general practice at the University of Oslo founded in November 1968. From 1969 to 1994 he was professor in General Practice/Family Medicine. He became professor number four in the discipline. 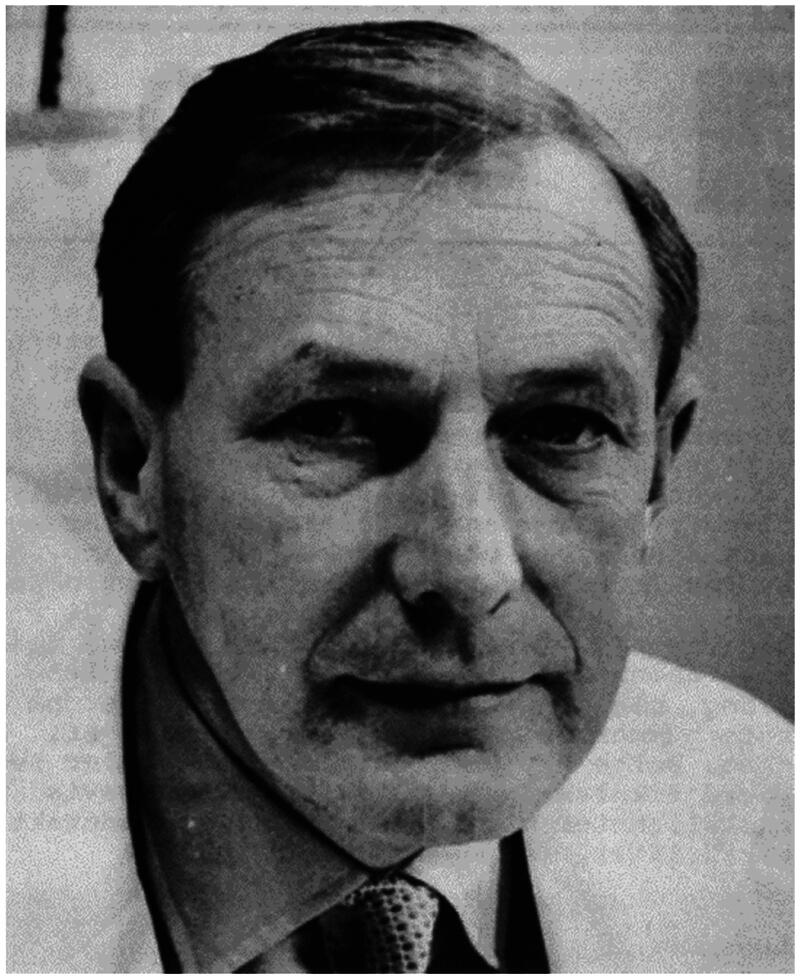 Alternative portrait of Borchgrevink (dated around 1968).

In 1967, the NMA succeeded in negotiations with the government to establish a fund for vocational training and continuing medical education. The NMA decided that an explicit objective for the fund was to fund an Institute of General Practice at the University of Oslo. The NMA then offered the university for free an Institute of GP/FM including all costs, professor’s salary for five years, and with a teaching practice. That was an offer hard to refuse and it was accepted both by the university and the Norwegian government. Because there were no academically qualified GPs in the country, the professor had to be recruited from another branch in medicine aiming to ‘convert’ the person to family medicine. Christian F. Borchgrevink (b. 1924) (Figure 4) specialist in internal medicine holding a Ph.D in hematology, received the professorship. It is part of the history that he had just been offered a professorship in internal medicine – which he refused. Before internal medicine, he had been working one year as a GP on the west-coast of Norway, and thereafter three years in Indonesia as a consultant for the WHO. The opening ceremony for the new institute in Oslo was on Nov. 27. 1968 [23], just a few months after Ian McWhinney had become professor at Western University in Ontario, Canada. The University of Oslo thus became number four in the world and first among the Nordic countries to have a GP/FM institute with a professor in the discipline.

In 1968, the NMA also gave a similar offer to the University of Bergen. In Bergen, a new building first had to be put into place, and the Institute of general practice there was founded in April 1972 [24].

The main priority for the new department in Oslo was on teaching for medical students. As there were no space for family medicine in the curriculum, teaching had to be optional the first years. Gradually, step by step, session by session, general practice teaching was expanded until today where GP/FM is one out of three main clinical disciplines (along with internal medicine and surgery) in the curriculum.

Borchgrevink played a central role in the professional development of family medicine in Norway during the 1970- and 1980-ies. He was one of the founding fathers of the Norwegian College for General Practitioners (Now: The Norwegian Association for General Practice) where he served as the first chairman 1983–1985. He was editor-in-chief of the Scandinavian Journal of Primary Health Care 1989–1996. He has extensive writing himself on various medical topics. Borchgrevink also worked internationally, among others by supporting development of primary health care services in northern Portugal during the late 1970-ies. After 25 years in position, professor Borchgrevink stepped down from the chair and retired in 1994.

At a seminar in 2009 about the development of Norwegian general practice to become an academic discipline Borchgrevink was one of the participants. Here, previous professor in GP/FM in Bergen, Per Fugelli (1943–2017), said to him that *‘You were … a shaping force in the early period, a creator of excitement, a builder, and a magnet for young people with greedy minds who saw that here it was possible to enter a new medical profession. You were the leader who stepped forward with bold courage. As they say in North Korea: You were our sun!’* [21]

Professor Borchgrevink was an important facilitator for the development of academic general practice both in Norway and in the Nordic countries. Almost to the age of 90, Borchgrevink still supervised GPs doing research in their practices. Among the first four professors in General Practice in the world, Christian F Borchgrevink is the only one still alive. By March 2024, he is 99, hopefully he will turn 100 later this year.

In 1975, Norway had professors in general practice/family medicine at all four medical schools in the country. In 1986, GP/FM was recognized as a medical specialty with a defined training program. A comprehensive Norwegian textbook in general practice was published in 1997 (latest edition in 2023) [25]. The textbook has also been published in Swedish, Danish and Estonian.

Despite a successful development within academic general practice during more than half a century, Norwegian general practice is presently facing challenges due to declining recruitment, increasing workload and underfunding.

## Professor Scott’s definition of general practice – still relevant

While he still was the only general practice professor in the world, Richard Scott gave the annual James Mackenzie lecture in 1964 at the University of Edinburgh. In the lecture, ‘Medicine in society’, he argued that GPs should be considered as specialists in anti-specialism [26]:

Since general practice itself advances by taking over from time to time some of the skills and techniques of the specialist, how can we ensure that the general practitioner does not respond negatively to these new challenges by himself becoming simply another specialist cast in the same mould - but adopts instead a more positive approach and becomes a specialist in anti-specialism?

He also gave a short and concise definition of general practice:

…a purely functional definition of general practice as being that sector of medical care in which the patient enjoys direct and continuing access to his own doctor. These two factors, direct access and continuity of access, are the only essential characteristics of general practice. Unless they are preserved, general practice will disappear.

His words are indeed relevant for the current challenges facing general practice today. The issue of personal continuity in the GP-patient relationship is about much more than just ideology. Almost twenty years ago, Barbara Starfield (1932–2011), professor of Health Policy and Management at the Johns Hopkins Bloomberg School of Public Health in the US, demonstrated that areas with a better primary care had better health outcomes, including total mortality rates, heart disease mortality rates, infant mortality, and earlier detection of cancers [27]. Recent research from Norway has substantiated that continuity of care in general practice has profound impacts on both health care use, morbidity- and mortality-rates [28]. It is simply better for both the society, for population health and for the individual patient.

Like medicine in general, general practice must also adapt to changes in society – the challenge is to do so without violating the essential core values of GP/FM [29].

## From practice to professorships – still a continuous work

The academic development of general practice may be characterized as an emancipatory process. An evolvement from a broad and unorganized field of medical practice into a medical profession based on principles, research, and training. The process required inspirational leadership, and in the early phase this was guided by academic pioneers. Efforts were required for GP/FM to be accepted as an independent discipline within universities during the 1960s. Both in Scotland and Norway, external funding was needed to pay for the first university chairs for the profession. Both in Utrecht and Ontario, the universities invested in the realization of professorships – which was justified by the extraordinary professional vision and expertise of the candidates.

**Figure 5.**
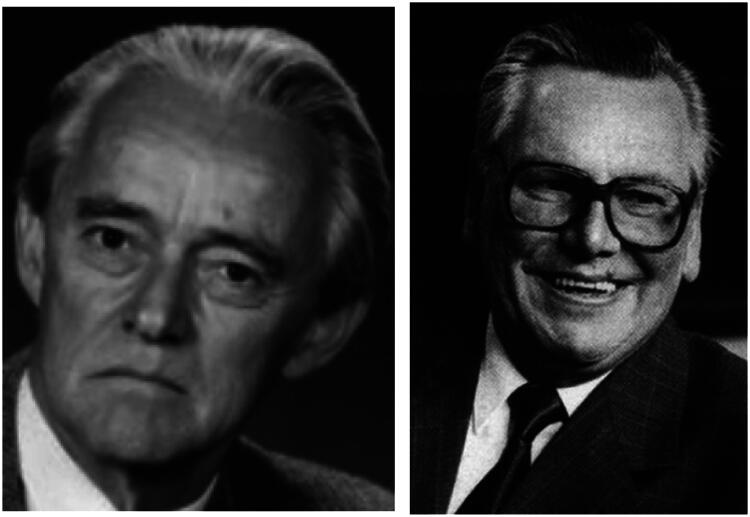
The first general practice professors in Denmark and Sweden: Left: Paul Backer (1927–1995), professor at the University of Copenhagen in 1974. Right: Bengt Scherstén (1929–2009), professor at Lund University (Dalby) in January 1981.

**Figure 6.**
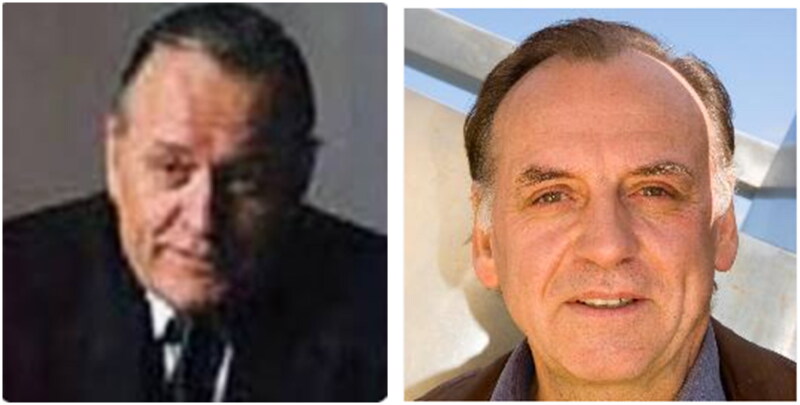
The first general practice professors in Finland and Iceland. Pertti Vilhelm Kekki (1940–) became acting professor in 1981 and professor in 1984 at the University of Helsinki. Johann Agust Sigurdsson (1948–) professor at the University of Iceland in Reykjavik since 1991.

Apart from Norway, the first GP/FM professorships in the other Nordic countries were established in 1974 (Denmark), in 1981 (Sweden and Finland), and 1991 (Iceland) (Figures 5–6). Today, all medical schools in the Nordic countries have professors in GP/FM.

**Figure 7.**
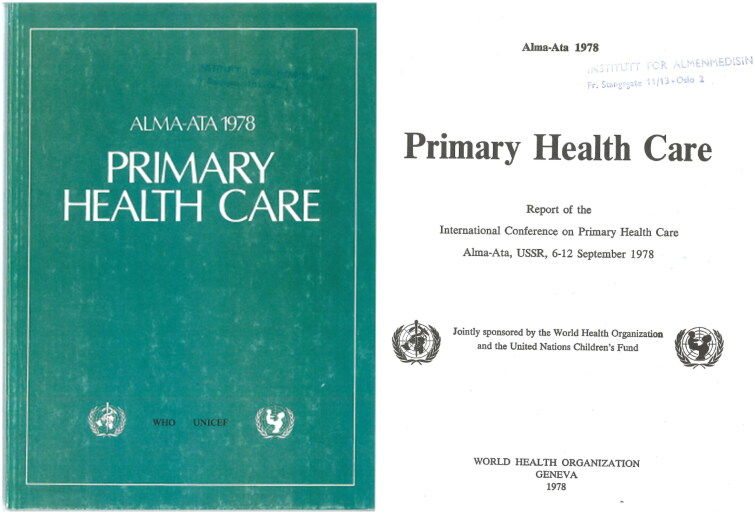
The Alma-Ata declaration (1978) represented an important step forward for recognizing the importance of primary care as the foundation for a well-functioning health care system in all countries in the world.

The global recognition of GP/FM to be an essential part of the medical education was also influenced by the 1978 Alma-Ata declaration by the World Health Organization (Figure 7) [30]. The declaration stated that primary health care should be the hub of the health systems in all countries.

However, even today (2024), many medical schools in Europe do still not teach GP/FM. In a survey from 2013 which included 259 medical schools in 39 European countries, 35 (13.5%) universities located in 12 different countries reported that they had no GP/FM curriculum [31]. This applied especially to universities in eastern and southern parts of Europe. But even high ranked universities in the US like Harvard, Stanford, and Johns Hopkins also lack separate family medicine departments and general practice programs in their medical schools [32].

By year 2024, the further development of general practice/family medicine as an academic discipline is still an ongoing and at times challenging process.
